# Hospital and Institutional News

**Published:** 1918-03-09

**Authors:** 


### HOSPITAL AND INSTITUTIONAL NEWS.

### THE RETIREMENT OF SIR ALFRED KEOGH.

Few men, we imagine, have more justly earned the thanks of the nation than Sir Alfred Keogh, whose retirement from the Director-Generalship of the Army Medical Service was made the occasion of a crowded and enthusiastic meeting in his honour at the Imperial College of Science and Technology last week, the rectorship of which he has now resumed after nearly four years' absence upon military duties. (See The Hospital, January 19, p. 333.) Mr. A. H. Dyke-Acland, in moving a resolution expressing pride in the distinguished services rendered by Sir Alfred, expressed his gratification that he is able to resume his work at the College at a time when its future importance in relation to the industries of the Empire is more fully recognised than ever before. The amazingly small death-rate from typhoid in our armies at the present day is due in no small measure to his influence and energy. In the days of Napoleon 97 per cent. of the deaths in war were due to disease, in the South African campaign 63. Now, in all the present theatres of war, the percentage from this cause does not rise above 4. No better justification could be advanced for the continuance of the Army School of Sanitation, where combatant officers undergo courses of instruction, and in assessing Sir Alfred Keogh's merits it must be borne in mind that it was he who was responsible for its inception in the face of much bitter and ignorant opposition. He was a member of the Brodrick Committee, which sat after the South African War to consider the reorganisation of the Army Medical Service, and from that proceeded to the Deputy-, and subsequently Directorship-Generalship, of the Service from which he is now retiring. We rejoice to record that His Majesty has appointed Sir Alfred to be a Companion of Honour for his services in connection with the war.

### THE TORPEDOING OF THE "GLENART CASTLE."

Yet another has been added to the lengthy calendar of crimes that humanity has to record against the German nation. The sinking of the *Rewa* is not two months old, and though fortunately on that occasion the loss of life was trivial in comparison to this, the dastardly motive remains the same. The *Glenart Castle* had complied with all the regulations of the Hague Convention. Red and green lights were carried, and she bore all the distinguishing marks of a hospital ship, nor was she even within the "barred zone," as delimited by the Germans themselves in an official statement issued on January 25 of last year, within which all hospital ships were to be sunk at sight. There is, therefore, no possible pretext or excuse for the act, which, we fancy, even the Germans themselves will be unable to explain save as an exhibition of calculated brutality. Within the past twelve months no fewer than six other hospital ships have been similarly dealt with, the most notable instance being the *Asturias*, in which the total casualties in killed and injured amounted to seventy-two. The death-roll of the *Glenart Castle*, aggravated by the extremely adverse weather conditions, numbers no less than 150, a trifling addition, perhaps, to the total casualties of war, but a heavy crime to book when the final reckoning with our adversary is being made.

### HOSPITAL EXPANSION IN WEST HAM.

The receipt by Queen Mary's Hospital for the East End of a Royal Charter of Incorporation, which was received on December 17 last, has now been presented to the governors. Thanks to the charter, many future difficulties have been overcome, including certain legal points in connection with the recent change of name from the West Ham and Eastern General Hospital to Queen Mary's Hospital for the East End. The old title was considered too local, as can be understood from the fact that nearly £19,000 was spent last year by the hospital, to which no fewer than 21,161 casualties were brought. The hospital received over 110 victims from the terrible East End explosion alone. West Ham, the borough, is the seventh largest town in the country, and it has been decided to build a maternity block as soon as this is possible, with the £10,000 given by Mr. Charles Lyle, the hospital's chairman, as a memorial to his late wife, Margaret Lyle. In fact great extensions will be required in the near future if the hospital is to do its duty to the population which it serves. One interesting item in connection with the satisfactory income of last year may be mentioned: the splendid result of the appeal which raised £4,400. A new departure was made in inviting the comedian, Mr. George Robey, to issue the appeal. The experiment was amply justified, and Mr. Robey, by a concert at the Alhambra alone, raised £900. In token of the hospital's expansion and usefulness, £5,000 was granted by the King Edward's Hospital Fund, together with the following words of encouragement: "£1,000 in recognition and aid of the special efforts made to improve the financial position of the hospital." In view of the future it is good to learn that the freehold of the adjoining cottage property on both sides of the hospital has been acquired. What is wanted now is a larger building fund in order that the much-needed accommodation may be gradually made available.

### THE BRADFORD WAR MEMORIAL.

Mr. George Priestman and Mr. H. Behrens did a public service at the recent annual meeting of the Bradford Royal Infirmary in using the achievements of the hospital during the past year as an introduction to a weightier matter. The former was able to show that the department opened last April for cases of venereal disease was vigorous and efficient, and that there were 360 in-patients and 1,000 out-patients who had already been benefited by its agency. He also pointed out the increase in the number of accident cases, mostly from munition works. He then remarked on the steady increase in the number and amount of annual subscriptions. The vigour of the work and the increased public interest awakened by it having been shown, Mr. Behrens followed and put his finger on a weak point. At the end of the war something should be done for the infirmary. He reminded the audience that a large piece of land had been taken on which to build a new hospital, but the money had not been forthcoming, nor did he think that the wealthy or the workpeople had yet done their share. What fitter memorial could there be than a new hospital? We hope that his hint will be taken, and now that the current of public interest is so healthy in Bradford opportunity should be taken to complete a scheme which has, unfortunately, been allowed to hang fire.

### BRITISH HOSPITALS ASSOCIATION.

The annual meeting of this Association will take place on Friday, March 15, at St. Thomas's Hospital. Lord Sandhurst, the President of the Association, will be in the chair. Preceding the annual meeting the Association will hold a conference on the question of the proposal to create a Ministry of Health and how it may affect the voluntary hospitals. The conference will be opened at 3.30 p.m. by an address by the Hon. Sir Arthur Stanley, G.B.E., C.B., Treasurer of St. Thomas's Hospital.

### THE SITE OF ST. MARK'S HOSPITAL.

The Lord Mayor, who was accompanied by the Sheriffs on his recent visit to St. Mark's Hospital, City Road, referred to the vacant site adjoining the institution. The site was now for sale, and he heartily commended the efforts being made by a few of the subscribers to secure it for the hospital. If this was to be accomplished, a balance of £4,000 would be required by April 1. If the site could not be purchased, and a factory were built upon it, that would be disastrous to the work of the hospital, for it would exclude light, air, and quiet. Mr. F. Swinford Edwards, F.R.C.S., Mr. Thomas Mark Merriman, Dr. Oxford, and Mr. F. Lockhart Mummery all referred in their speeches on the same occasion to the great opportunity now presented to extend and improve the hospital's work by the acquisition of the vacant site.

### ARUNDEL HOSPITAL: PAST AND PRESENT.

The late reopening by the Duchess of Norfolk of the Arundel and District Cottage Hospital is a small sign of the times. The Duchess, who founded the institution, and before it was reconstituted was responsible for its control, explained in a frank and engaging speech that the estate could no longer be responsible for a very uncertain liability. Great changes had occurred, and when the estate had had to pay the large debt of £400 on the hospital at the end of a year reconstitution was inevitable. She was glad that the townspeople appreciated the circumstances of the change, and had already voluntarily promised a weekly sum in support of the hospital. She hoped that they would agree that the estate was doing its share in giving a certain fixed sum, and in limiting its responsibilities to that. They must all work together to make it a success, and they might be sure that she would maintain her interest in it. This event, which was inevitable, has a certain historic interest. It shows that the great estates of England under modern conditions have to lose something of their personal and patriarchal character. They, too, alas! have now to be run on more businesslike and less personal lines. This is the price of a hospital which is now not one of the charities of an estate, but the property of the people upon it.

### A SUCCESSFUL COTTAGE HOSPITAL.

Lieut.-Col. Atkins was heartily welcomed back to civil life in presiding over the twenty-seventh annual meeting of the Hinckley (Leicestershire) Cottage Hospital. The hon. secretary, Mr. A. W. Crofts, reported that 175 in-patients had been received during the year, whose average stay was sixteen days. Out-patients continue to attend in larger numbers, 155 new patients and 1,437 attendances being recorded. Complimentary reference was made to Miss Edgington, the matron, and to Nurse Earp, who as district nurse had performed most useful work under the supervision of a ladies' committee. The hon. treasurer, Mr. F. Green, submitted the most successful financial report for twenty years. Annual subscriptions and workpeople's contributions both show an increase. Donations were larger and more representative. The increased workpeople's contributions were particularly satisfactory, and the augmented subscription list was almost entirely due to the activity of Mr. Jee, the new honorary financial treasurer.

### A NEW HOSPITAL FOR NORTH CHINA.

To commemorate the work of the late Rev. E. H. Mosse, whose son, Dr. F. H. Mosse, now on military medical service in Egypt, has determined to become a medical misssionary, the Society for the Propagation of the Gospel has decided to establish a memorial hospital in North China to be called after the father's name. Since this district is to be the centre of Dr. F. H. Mosse's future work it is possible that he may become attached to the new institution. To build the new hospital £3,000 is required, and the society is hoping to raise another £2,000 for its endowment.

### NEWS FROM PALESTINE.

The Syria and Palestine Relief Fund has furnished us with some interesting details of the work accomplished at the Khan Unis Relief Camp Hospital. Dr. Lloyd explains that in all the relief work he has met no one who has been fighting against us. A great rush of wounded civilians immediately following the rapid Allied advance for the time thrust into the background the normal hospital and dispensary work normally carried on in the twenty-odd tents at Dr. Lloyd's disposal, tucked away in the sand dunes at the south-east corner of the Mediterranean, within sight and sound of the sea. Ambulances and stretchers began to arrive in batches, and the story was always "First the British shelled our village when the Turks were in it, and the Turks shelled it when the British were in it." Most of the wounded were women and children. Army hospitals have little room for civilian men, much less for women and children, and it requires little imagination to guess what a hospital for civilians meant to the Syrians. The Relief Fund Orphanage at Jaffa does what it can to find a home for children whose parents have been killed. One boy of eleven had both hands blown off. But for the Relief Fund his fate in life would be that of a professional beggar. Amputations of arms and legs are common, and the operations performed ran into scores. Clothing had to be provided for many refugees, and for the discharged wounded, the latter needing a complete outfit in which to face the journey home. Hundreds of blankets were distributed among the convalescents and the shifting population of the camp, who often saved up the blankets so as to form a kind of great-coat which might be worn either day or night, or both. Rations were provided by the military authorities, but, as has been insisted before, these rations need to be supplemented from the Fund. For such further news as has come through application should be made to the office of the Syria and Palestine Relief Fund, at 110 Victoria Street, S.W.

### AN OBJECT-LESSON IN PERSISTENCE.

Secretaries are having an object-lesson at Cardiff in the policy of persistence, since Mr. Leonard Rea's letters to the Cardiff papers form a sequence, which is a welcome addition to their columns, and has certainly been productive to the hospital. We have noted the series in some detail because the word "persistence" does not impress its lesson as clearly as each step in turn by which this persistence has been maintained. The letters fall into two classes. The first deals with any incident which may be used to illuminate the hospital's work or needs, a word heard at a street corner, a sentence in a letter of thanks, the comparison suggested by the waiting queue outside a cinematograph theatre, and so on. The second class of letter deals with the actual cost and number of patients received from each district, and compares with it the total contributions from the residents there. Like most fruitful ideas, this one is old. What is new is the freshness with which the idea is being presented. A chance allusion, as we noted at the time, led the places beginning with the letter P to be tabulated in this way. This suggested running through the alphabet. The result of this method has been to remind the public that the districts whose contributions compare best with the cost of the patients sent by them are those which hold a penny-a-week collection. In truth, this plan never fails when carried out vigorously with continuous pressure. The Prince of Wales' visit coincided with the flood tide of energy at King Edward VII.'s Hospital. These things come neither by chance nor sudden endeavour. They are the rewards, and the proofs, of a long record of work. It was hard luck on Colonel Bruce Vaughan to have been prevented by illness from attending. But what a tribute to him is the continuance of the vigorous policy which he inspired!

### THE IMMENSE VALUE OF GIFTS IN KIND.

It is such a trouble to start a collection of gifts in kind that their value receives too often only perfunctory acknowledgment. To the recent striking success achieved at Cambridge from such gifts we can now add another example from the Victoria Hospital, Keighley. Mr. J. E. Haggas, the hospital's treasurer, lately declared the small amount of money spent on food at the hospital was most surprising. Between 1916 and 1917 the cost of food had increased only 2d. per head, notwithstanding the advance in prices. This was accounted for, he added, by the generosity of many friends of the wounded, who had sent in presents of food. Mr. Haggas fears that this feat of economy cannot be repeated. We wonder. In these days of substitutes and revised diets, the value of vegetables and fruit, to say nothing of eggs and cheese (if cheese exists), cannot be over-estimated. The potato is now a staple. There is still plenty of opportunity for collecting gifts in kind, and the circumstances of the hour make them doubly to be sought for.

### DEATH OF A HOSPITAL SECRETARY.

It is with regret that we record the sudden death of Mr. W. C. Lewis, secretary of the West Kent General Hospital. Mr. Lewis, who had held the post for seventeen years, leaves a widow and family, to whom the board has sent a token of its appreciation of his "untiring services."

### CHELTENHAM HOSPITAL'S NEW SECRETARY.

The board of management of the Cheltenham General Hospital have appointed Mr. J. B. Stevenson to the post of secretary, vacant on the resignation of Mr. W. H. Head. Mr. Stevenson, who is twenty-four years of age, is a son of the general superintendent and secretary of the Devonshire Hospital, Buxton, and is permanently exempt from military service on medical grounds. The new secretary has had six years' hospital experience at the Devonshire Hospital, where he is at present assistant-secretary under his father, and prior to his appointment to the latter post he was patients' registrar and clerk in the steward's department.

### THE SALARIES OF MEDICAL SUPERINTENDENTS.

In July 1914 the General Purposes Committee of the Metropolitan Asylums Board decided that in future a minimum and maximum salary should be assigned to the post of medical superintendent of each of the Board's hospitals. The committee, in return for the surrender of certain emoluments, proposed various increases of salary, but on these the Local Government Board has hitherto given no decision. At a meeting of the Finance Committee on February 12 it was agreed that, in fairness, the individual superintendents concerned should now receive the salaries and emoluments which they would have been enjoying if the managers' proposals in 1914 had been officially sanctioned. These proposals, which showed a necessary advance on the scheme of 1908, recommended that the existing superintendents should receive a maximum salary of £875 per annum, with an unfurnished house or quarters. The salary was to include £25 a year in lieu of washing. Should these proposals be sanctioned by the Local Government Board, and surely further delay on its part would be inexcusable, the Principal Medical Officer of the Asylums Board, Dr. Herbert E. Cuff, O.B.E., M.D., will be placed in a disadvantageous position in regard to that of the medical superintendents. The managers therefore propose that the above scheme shall include an increase of his salary by £100 a year. This will make his salary £1,000 per annum, which is his due in the light of the proposed changes, apart from his twenty-five years' service with the Asylums Board.

### MILK EXTRAVAGANCE IN OFFICERS' HOSPITALS.

The Army authorities have just issued a scale of rations for the guidance of those in charge of military hospitals, one of a value of 2,700 calories per patient. Stringent orders have also been promulgated with regard to milk, the consumption of which, especially in officers' hospitals, is far too high. Cocoa, it is pointed out, forms an excellent substitute for the mid-morning glass of milk and for the supper-time hot milk as well, whilst the taking of milk as a drink in officers' mess must also be curtailed.

### THE FUTURE OF THE MAIMED SOLDIER.

Some remarkable statements concerning the future of the disabled soldier, as actually observed on his return to civil life, were made at the recent meeting at the Mansion House on behalf of Queen Mary's Convalescent Auxiliary Hospital, Roehampton. The speakers included Lady Falmouth and the senior surgeon at the hospital, Colonel Openshaw. Lady Falmouth stated that thousands of the men treated at Roehampton had returned to civil work at higher salaries than they had ever earned before. This is a startling, if highly gratifying, statement. The same remark applies to Colonel Openshaw's dictum, in pleading for the permanence of his hospital, that a man lives longer if his leg has been amputated. In both cases it would be interesting to have the figures which support these generalisations. We should hardly have thought that the war had lasted long enough to justify Colonel Openshaw's assertion, or that statistics of the longevity of cases of amputation had been satisfactorily collected before the war. In regard to Lady Falmouth's remark the question arises if the posts found for the men of whom she speaks are likely to be permanent, or how far the posts and the salaries are themselves dependent on the present condition of the labour market.

### DISCHARGED SOLDIERS AS ATTENDANTS.

As we have already reported, the Birmingham Board of Guardians has been considering the training of discharged sailors and soldiers as attendants at the asylum. The committee asked to deal with the matter reports that, after consultation with the chief medical officer, the matron of Monyhull Colony, and the head officials of the three houses, the proposal is approved. Not only would the training of the discharged soldiers be useful, but the Union would benefit from such a supply of men available for appointments under the Board. The committee's scheme, which may have six months' trial, lays down that six months' instruction should be given in first-aid, the supervision and sick nursing of male mental cases, and in the utilisation on the land of the services of male inmates of sufficient capacity. Instruction to be given by the paid attendants. Lectures will also be held. Not more than six attendants are to be in training at the same time at any one institution; the period of training is to be four months. Discharged sailors and soldiers who show aptitude in the training and are pronounced proficient will be eligible for appointment to vacancies at the Guardians' institutions, or assisted to obtain appointments elsewhere. While under training the men will be non-resident and not entitled to any emoluments, except overalls. The co-operation of the Local Statutory Pensions Committee will be sought, and application made for the approval of the Ministry of Pensions, in order that the prescribed allowances may be made to discharged sailors and soldiers undergoing training.

### RATIONS IN SANATORIA

The President of the Local Government Board has considered with the Food Controller the position of sanatoria in relation to compulsory rationing. For sanatoria the Board has formulated special dietaries, and these scales have been temporarily approved by the Food Controller, and will apply only to patients actually suffering from tuberculosis. The officers of the sanatoria must, of course, comply with the general scale of rations prescribed by the Ministry of Food unless they are suffering from tuberculosis. The scale for children under ten will be three-fifths of the corresponding scale for the adult woman. The actual dietaries we give on page 492.

### THIS WEEK'S DRUG MARKET.

The upward tendency of prices continues to be the main feature of the drug market. The difficulty of obtaining supplies of potassium and sodium bromides is more pronounced, and a further rise in quotations is probable. The demand for saccharin is increasing, and the high prices are maintained; Government control of this article is still being discussed. Chloral hydrate is in good demand, and the price is rather higher. Only small supplies of lithia carbonate are available, and prices have an upward tendency. Acetanilide continues scarce. The stock in London of American peppermint oil is much reduced, and holders are asking higher rates. Sugar of milk has again substantially advanced in price. Senega root is dearer. Quotations for English distilled clove oil have been raised. Apomorphine is dearer.

